# The Dental Filling

**Published:** 1896-11

**Authors:** Joseph Head

**Affiliations:** Philadelphia, Pa.


THE
AMERICAN JOURNAL
—OF—
DENTAL SCIENCE.

VOL. XXX. BALTIMORE, NOVEMBER, 1896. No. 7.

ARTICLE I.

### THE DENTAL FILLING.

BY JOSEPH HEAD, D. D. S., PHILADELPHIA, PA.

For the last fifty years dentists have declared that a filling must be a water-tight, forgetting that the tooth itself is thoroughly pervious to moisture, and also forgetting that many fillings which admittedly leak keep on preserving the tooth-structure indefinitely. Jack has put in soft foil fillings, under water, that have done good service for years. Elliott's observations concerning the universal leakage of amalgam plugs are verified by the daily experience of each dentist, and yet who will say that in spite of leakage amalgam does not save the teeth well?

Drop a fresh tooth, filled with gutta-percha, into aniline ink, and at the end of five minutes the entire cavity under the filling will be stained.

The oxychloride of zinc and oxyphosphate of zinc are permeable to moisture; nevertheless cement and gutta-percha are indispensable to those operators who would serve the best interests of their patients.

Cohesive gold and tin are claimed by their admirers to be non-leaking materials, but when a tin or cohesive gold filling is first carefully cleansed and then melted, a decided order of burnt organic material will be perceptible, which would seem to emanate from the interior of the metal. In the light of this fact further proof is necessary before cohesive gold and tin can be said to positively seal the cavity margins, And if they should be proven to absolutely seal such margins, the mere fact that the other materials frequently preserved teeth would be conclusive evidence that leakage of moisture in itself is not a serious objection.

The destructive electro-chemical action of filling-materials on the teeth, once so strongly advocated by Chase, has been proven by Miller to be untrustworthy; and if Miller's experiments had not been so conclusive, the daily dental operations, where tin and gold are advantageously combined, would of themselves, as years go by, prove the electro-chemical action in the human mouth quite harmless.

That such currents do exist at times no one who has put an amalgam filling in an acid mouth containing gold can for a moment doubt. And it is certainly true that such currents are invariably accompanied by chemical dissolution; but practically no harm results, as an insulatory coat soon forms on the amalgam which effectually puts a stop to any serious corrosion. Instead of saying that an efficient filling should be absolutely water-tight, and should have no electro-chemical action on the teeth, might it not be said that the perfect filling will exclude bacteria from the cavity as thoroughly as would normal enamel; in fact, that it will exclude them absolutely?

Let us see if we have such a filling-material. Oxychloride and oxyphosphate of zinc leak bacteria, the proof being as follows: Hollow balls of oxychloride of zinc and hollow balls of oxyphosphate of zinc were thoroughly sterilized, and then dropped in a solution of bouillon that had been inoculated with a decayed tooth. At the end of five days they were opened. The bouillon had filtered through the substance. The bouillon found within was swarming with bacteria.

Gutta-percha was tested as follows: Several old cuspid teeth of dense structure were drilled through from end to end, the pulp-canal being eradicated. With proper precautions these were sterilized and filled at each end with gutta-percha, a small pellet of cotton soaked in sterilized bouillon being left inside. These were subjected to steam heat for an hour, five separate times, an interval of a day elapsing between each heating. They were then placed in tainted bouillon. At the end of five days they were examined, and the cotton soaked by broth was found to contain cocci, piplocci, streptococci, and staphylococci. The reports of these two experiments are given at full length in the June number of the *International Dental Journal*. The gutta percha test is not conclusive, as Miller has shown that bacteria may very occasionally penetrate the normal destinal tubules; but at least the experiment would seem to indicate that gutta-percha could not keep them out, which is the real point at issue.

That valid amalgam fillings at times leak bacteria as well as moisture, no experienced practitioner will deny.

Tin and cohesive gold are the only materials that may exclude bacteria. That they do exclude them is yet to be proved. Soft-foil fillings have been picked out from cavities by the explorer in a pulpy, evil-smelling state, to all appearances full of bacteria, and yet the dentine beneath has been found firm and sound.

It is a most astonishing fact that soft-foil fillings may be soft and mushy without the least harm to the protected cavity; while, if cohesive foil fillings are soft or defective on the edge, decay almost invariably sets in. Miller claims that soft foil has a slightly antiseptic action, which is lost in annealing. It might be said that a not too dense filling would be less likely to dangerously expand or contract under the action of cold or hot drinks. But these explanations do not seem sufficient. The questions raised by these facts cannot as yet be satisfactorily answered, and I hope some experiments on this subject, which are now in process, will give more positive light.

How is it that fillings can leak bacteria and still preserve the teeth? The fundamental principles of dentistry would seem to be shaken and antisepsis set at naught did we not remember that the quantity of bacteria, and the presence or absence of a suitable culture, is a most important factor in the process of decay.

Every man in a state of health, passing through the city, may safely inhale a limited number of small-pox germs, as the tissues and white blood-corpuscles will destroy them.

If, however, he works in a hospital, or wears the clothing of a small-pcx patient, the number of germs taken into the body overcomes the hygienic police force, and the man falls sick. The same principle applies to tooth-filling. The tooth can resist the onslaught of a few bacteria, and, it would seem, destroy them, as is shown in the case of mummified block decay. Moreover, many bacteria need the presence of air, and all need a constant supply of food. The fillings tend to exclude both of these essentials for bacteriological propagation.

In spite of the fact that a useful filling may leak micro-organisms, there should be no pains spared to adapt each plug as accurately as possible. The more perfectly a filling excludes bacteria from a sterilized cavity, the more certain the chances of permanent success.

And after all has been said against filling-materials and defective conditions of the saliva, it seems probable that the great majority of our failures arise either from hasty preparation of the cavity or unskilful manipulation.

In my opinion, a gold filling, either of soft or cohesive foil, if it has perfect adaptation to good enamel-edges, will preserve the tooth as absolutely as if the original enamel remained dense and undecalcified. A filling at beast can only restore the tooth to its original condition of perfection, and if the acid and bacteria; which originally created decay, should attack it again, there is no reason why the tooth-structure should not disintegrate a second time. This is an unanswerable excuse, and may convince the patient many times, but if decay recur too often in the same place, the coincidence is most unfortunate for the dentist.

I am well aware, from my varied experience in dental meetings, that there are numerous practitioners who always do absolutely perfect work; whose hands never tremble from fatigue at the end of a long day; whose weary eyes never by any possibility overlook small concealed portions of decay that ought to be removed. These practitioners will not need the suggestion I am going to make, because when no fault can exist no precaution is necessary. But to that large and most useful class of dentists who, in spite of a personal element of error, engage to relieve pain, preserve teeth, and make mouths wholesome —to them I would say: After the gold filling, approximal or crown, is inserted to the best of their ability, let them polish it down almost flush with the enamel-edges, and rub in thoroughly a mush of amalgam. When the patient returns, they can polish all the amalgam off and finish the filling, which will look untarnished and resemble any ordinary filling except in one particular. Should decay attack its borders, the conscience of the dentist may be quite clear.

Of course the amalgam that fills up any small fissure will shrink, while hot and cold drinks may cause the gold to expand and contract from the cavity walls. But objections to gold fillings are now out of date. Gold for upward of fifty years has prevented decay. The teeth can successfully antagonize a small number of bacteria, and it is my opinion and my experience that the edges of a gold filling, guarded with amalgam as described, will successfully prohibit the dangerous entrance of micro-organisms.—*Cosmos.*

